# Referral of Patients for Surgical Aortic Valve Replacement before and after Introduction of the Transcatheter Aortic Valve Implantation—Changing Patterns of Preoperative Characteristics and Volume and Postoperative Outcome

**DOI:** 10.3390/jcdd10050223

**Published:** 2023-05-22

**Authors:** Wilhelm Mistiaen

**Affiliations:** 1 Faculty of Medicine & Health Sciences, University of Antwerp, 2610 Antwerp, Belgium; wilhelm.mistiaen@uantwerpen.be; Tel.: +32-38305002; 2 Department of Health and Sciences, Artesis-Plantijn University of Applied Sciences, 2000 Antwerp, Belgium

**Keywords:** transcatheter aortic valve implantation, surgical aortic valve replacement, risk score, mortality

## Abstract

Transcatheter aortic valve implantation (TAVI) was first presented in 2002 as a case report. Randomized controlled trials showed that TAVI could serve as an alternative for surgical aortic valve replacement (SAVR) in high-risk patients. While the indications for TAVI have expanded into low-risk groups, favorable results of SAVR in elderly showed an increase in application of surgical treatment in this age category. This review aims to explore the effect of the introduction of TAVI in the referral for SAVR with respect to volume, patient profile, early outcome, and use of mechanical heart valves. Results show that the volume of SAVR has increased in several cardiac centers. In a small minority of series, age and risk score of the referred patients also increased. In most of the series, early mortality rate reduced. These findings, however are not universal. Different management policies could be responsible for this observation. Moreover, some patients in whom aortic valve replacement in whatever form is indicated still do not receive adequate treatment. This can be due to several reasons. Heart teams consisting of interventional cardiologists and cardiac surgeons should become a universal approach in order to minimize the number of untreated patients.

## 1. Introduction

Symptomatic aortic valve disease, if left untreated, has a high mortality rate [1,2]. Its incidence increases with age and treatment in elderly is more challenging [3]. Surgery was the only treatment available in our own valve series between 1986 and 2006, during which 1000 patients were referred for surgical aortic valve replacement (SAVR) with a biologic heart valve prosthesis (BHV). In this period, the numbers, the age, and the comorbid conditions increased over time, while early mortality and postoperative cardiovascular complications did not. Only non-cardiac complications increased significantly. These data were collected up to two years before the introduction of transcatheter aortic valve implantation or TAVI [4]. TAVI was first described in a case in 2002 [5] and its value has been proven for patients not suitable for SAVR [6]. However, earlier obtained favorable postoperative results in octogenarians in terms of survival and of quality of life [7,8] inspired the expansion of the indication of SAVR in elderly patients. This age group is known to have more comorbid conditions, but in selected patients, results can be as good as in younger patients [9]. SAVR, therefore, could continue to have a place in the treatment of aortic valve disease, even in the era when TAVI is expanded in patients with medium and even low risk [1,10,11]. The question arises about the extent to which SAVR has a place since it is major surgery with a long rehabilitation time. This is important since there is a negative relationship between volume and outcomes after cardiac surgery [12]. The research questions in this systematic review are:

- What is the volume of patient series published over time (before v. after the introduction of TAVI)?
- What are the time spans involved?
- What is the effect of the introduction of TAVI on (a) age, (b) risk scores, (c) use of mechanical valves, and (d) early mortality?
- Is any information available on untreated patients with aortic valve stenosis?

## 2. Materials and Methods

The following term was used to search the Web Of Science database in March 2023: “Aortic valve replacement AND surgery AND era”. Using simple search terms, a broad sweep could be made. This resulted in 539 hits. There were no filters for time limits, since TAVI is a relatively new procedure. The main inclusion criterion was the comparison of SAVR with or without CABG over time (per year or per interval of a few years) before v. after the introduction of TAVI. After exclusion of reviews, overviews or comments, editorials, letters, abstracts, and case reports, 177 references remained. After excluding other topics based on title and abstract (absence of trends in time, or main focus on endocarditis, bridging balloon aortic valvotomy, aortic valve repair, low-flow low-gradient, aortic arch, Ross procedure, childhood, procedures on any other valves, cardiopulmonary bypass time, readmission, race and gender, natural history of aortic stenosis, on pacemaker implant), 13 full articles remained in the first round [1,2,3,13,14,15,16,17,18,19,20,21,22]. Twenty-two articles were identified as secondary references [23,24,25,26,27,28,29,30,31,32,33,34,35,36,37,38,39,40,41,42,43,44]. The screening was performed by the only author, without automation tools. All included series with findings of age, risk score, and early mortality after SAVR (as means ± standard deviation or median with interquartile range) were tabulated. The data for TAVI were outside the scope of this review. No ethical approval was needed.

## 3. Results

### 3.1. Type, Size, and Eras of Included Series

Table 1 shows the authors in alphabetical order with publication year, the design with respect to the investigated population (nationwide, regional, or monocentric), the year when the inclusion started or ended, and the type of intervention (isolated SAVR, SAVR combined with CABG, or pooled). All series were observational and retrospective in nature. In monocentric series, patient inclusion was consecutive, thereby minimizing bias. In one series, male and female patients were investigated separately [3]; in another, TAVI and non-TAVI centers were analyzed separately [35]. In three series [26,28,34], patient age was represented as age classes, not as mean or median. Fifteen series were nationwide and mostly of recent origin, i.e., published in 2017 or later [13,18,21,22,23,24,26,30,31,33,35,38,40,41,43]. Three series covered a region [1,19,25] and fourteen series were monocentric [2,3,14,15,16,17,20,27,28,32,34,36,37,42], and one series was derived from data from three centers [39]. These series covered a time span between 3 and 15 years and only two series started before 2000 [23,43]. Most of the nationwide series covered over 20,000 patients, while the 14 monocentric series varied from small series, including less than 100 patients [2] to almost 2000 patients [28]. Eighteen series studied isolated SAVR [2,3,14,15,17,18,19,20,22,23,26,27,31,34,35,37,39,40] and six series investigated isolated SAVR and SAVR with concomitant CABG separately [1,13,21,38,41,43]. This included one study which also made the comparison with minimally invasive SAVR [39]. Nine studies pooled isolated SAVR with SAVR with associated CABG [16,24,25,28,30,32,33,36,42].

### 3.2. Outcomes of the Included Series

Table 2 offers an overview of volume, age, risk scores, and 30-day mortality, at the start and the end of the inclusion. These data were directly retrieved from the included manuscripts. The differences between the pre and post TAVI introduction can be considered as a representation of an effect measure. However, different modes of representation within each of the included manuscripts makes comparison between the series difficult. Significant differences are given in **bold letter** type, with at least <0.05 and in many larger series < 0.001.

#### 3.2.1. Volume

Most series represented the volume as the raw number of referrals for SAVR. Two series [38,43] represented them as referrals per 100,000 of the general population. Three series represented the volume as annual referral [23,25,42]. Eleven nationwide [13,18,22,23,24,26,30,33,38,41,43], regional [44], and eleven smaller [3,14,16,20,25,28,29,32,36,39,42] series showed an increase in volume of SAVR. In one study [35], the volume increased only in hospitals that did not perform TAVI. Volume of SAVR did not change significantly in three series [1,27,34], while the volume was reduced in five series [17,19,21,31,40] and in one series in TAVI-performing hospitals, but not in other centers [35].

#### 3.2.2. Age

All series dealt with elderly patients undergoing SAVR. The mean or median age ranged between 65 and 83 years in the earlier eras, and between 67 and 85 years in the later eras. Eleven series documented an increase with at least one year in age over time [2,3,14,16,19,23,24,32,33,38,43]. In one series, age increased only in female patients, not in male patients [3]. A decrease in age with at least one year was documented in six series [3,15,17,18,21,35,38], while no significant change in age over the years was documented in another seven series [20,22,30,34,36,41]. One series, presenting age as classes, showed a decrease in octogenarian patients while those younger than 75 years increased with time [26], while in another series, this younger age group increased [28]. Two series showed bars [40] or curves [39] instead of means or medians, which make interpretation more difficult, but the results still indicated a lower use of SAVR in elderly over time. In spaces left blank, age was not separated as pre- v. post-TAVI era, and was not useful in this context. A universal risk score was not used. Therefore, this parameter is not tabulated and individual series cannot be compared. Temporal changes can be observed, however. An increase in risk score over the years was observed in nine series [17,20,22,23,28,30,33,38,43], and in one series, there was a drop after an initial rise [39]. In some series, this increase was more prominent in female patients [3] or in hospitals with high TAVI volume [35]. Some series described a temporal increase in specific conditions such as having a prior sternotomy [16,36], or diabetes, liver, or kidney disease [38]. A non-significant change in risk score over the years was observed in another six series [20,22,30,34,36,41]. A decrease in risk score was observed in five series [21,31,35,40,41]. In one series, the profile in patients’ comorbidity changed without affecting the risk score: referral was less for patients with vascular or pulmonary disease or high NYHA class [16].

#### 3.2.3. Risk Scores

A universal risk score was not used in the included series. Therefore, this parameter is not tabulated and comparison between series is not possible. Temporal changes within individual series can be observed, however.

An increase in risk score for SAVR over the years was observed in nine series [17,20,22,23,28,30,33,38,43], and in one series there was a drop in the score after an initial rise [39]. In one series investigating the gender effect on risk scores, this increase was more prominent in female patients [3]; in another series investigating the effect of TAVI volume, an increase in risk score was observed in hospitals with high TAVI volume [35]. Some series described a temporal increase in patients with specific conditions such as prior sternotomy [16,36], or diabetes, liver, or kidney disease [38], but without providing risk scores.

A decrease in risk score was observed in five series [21,31,35,40,41]. A non-significant change in risk score over the years was observed in another six series [20,22,30,34,36,41]. In one series, the preoperative profile in patients changed after the introduction of TAVI: referral decreased for patients with vascular or pulmonary disease or high NYHA class, but these changes did not significantly affect the risk score [16].

#### 3.2.4. Valve Type

The type of valve prosthesis was not always described. None of the series showed an increase in the use of mechanical heart valves over time. Only one study showed no significant change over time [26], while eleven series documented a decrease in use of these devices as a number or with a descending curve [1,18,22,23,25,30,33,40,41,42,43].

#### 3.2.5. Mortality

Short-term mortality after SAVR showed a decrease over time in sixteen series [13,16,18,20,21,23,26,27,28,30,33,35,38,40,41,43], in spite of an increase in risk scores in six series. This decrease was highest in hospitals with a high-volume TAVI program [24], but a decrease was also observed in hospitals without a TAVI program [35]. Three series reported an overall low mortality [3,37,39] and six series showed no major changes [24,31,32,34,36,37] or an indiscernible pattern [42]. The nationwide surveys showed that the best results were obtained in younger patients, although the improved outcome was also observed in older patients, with comorbid conditions [38,41] and with an increase in SAVR volume [18]. Non-elective SAVR was associated with increased mortality. Association of CABG did not affect the outcome [38], but this was not true for all series [33]. Improvement in hospital mortality after isolated SAVR was observed in both genders, and all age groups and risk classes [28], including high-age patients [21,40]. In one series, however, mortality in all risk groups for SAVR remained unchanged throughout time, although the expected mortality decreased and the observed/expected ratio increased [31]. Some series indicated that mortality for SAVR was not affected by the introduction of TAVI [36].

## 4. Discussion

The current overview shows that in most centers, regions, and nationwide registries, the volume of SAVR procedures increased after the introduction of TAVI. There are several reasons for this increase in SAVR procedures. First, with the ageing of the population, degenerative aortic valve disease can be expected to increase [3,25,34,41,45]. However, the use of any valve replacement technique could outpace the increase in patients, indicating that here has been an underuse in available treatment options in the past [33,45]. Underuse can be suspected since 30 to 40% of aortic valve patients do not undergo a valve procedure [45], something which was observed over a decade ago [46]. This underuse has serious consequences, since patients who receive only a medical treatment suffer more stroke, rehospitalization, and death after one year [47]. Second, the increase in the number of SAVR procedures is higher in centers with a TAVI program compared to those without such facilities [16,24,25,26,30,32,33,38]. This has also been labeled as a “halo-effect”. The availability of a TAVI program might increase referral of high-risk patients, who after examination by a heart team are still referred for surgery. Third, reimbursement policies differ widely. In some countries such as Germany and Switzerland, TAVI, expressed as rates per million population, has penetrated significantly compared to other countries such as the UK, Belgium, and some East European countries [48]. In Germany, the sum of valve procedures increased over time in all age classes, mostly in patients older than 80 years. TAVI even outpaced SAVR while surgical procedures decreased moderately [40]. This could be due to the reimbursement policy, which in Germany is higher for TAVI compared to SAVR [48,49]. In contrast, in Belgium, advice was given in the past to not reimburse symptomatic aortic valve patients for TAVI, even if these have serious comorbid conditions with high operative risk. According to these authors, these patients should be referred for SAVR and the TAVI procedure should be reserved for patients deemed inoperable for anatomical reasons [49]. This might be the explanation for the significant increase in SAVR after the introduction of TAVI that was observed in our own aortic valve series. The first 1000 patients underwent SAVR with a BHV in a time span of 20 years [4], while subsequently, 1500 patients received a SAVR in only 10 years, between 2008 and 2017. A preliminary analysis showed that these patients also had significantly more history of malignancy, a condition that might favor nowadays TAVI as treatment [50]. The interaction between the introduction of TAVI and the volume of SAVR might be complicated by the rule of the advisory panel to the Centers for Medicare & Medicaid Services that a TAVI center can only open if at least 50 open heart operations were performed in the prior year [17]. Moreover, the presence of a dedicated heart team is a prerequisite for coverage by Medicare [51]. In Belgium, comparable advice has been very strict from the start [49]. In some countries, patients over 80 years are automatically referred for TAVR [1]. In these cases, a reduction in the number of SAVR can be expected. The surgical volume could also decrease if the indication of TAVI is expanded to medium- and even low-risk patients [11].

One could expect that, with the availability of TAVI as a treatment option, the number of older and high-risk patients referred for SAVR would drop. However, a mixed picture can be derived from the included series: eleven series showed a significant increase in age over time, six series showed a significant decrease in age, and nine series showed no significant effect.

The drop in age of patients referred for isolated SAVR was higher in TAVI hospitals, compared to those without a TAVI program [35]. A cross-over between SAVR and TAVI might be responsible of this observation. In some series, an initial increase in referral of octogenarians for SAVR was followed by a decline [39]. The decrease in the number of SAVR patients was most obvious in octogenarians [40]. However, in one series the octogenarian group increased [28]. This is also our preliminary observation: not only did the overall number in our institution increase, but also the rate of octogenarians; we analyzed the outcome of 861 octogenarian patients who underwent SAVR between 1987 and 2007 [52]. They were a part of 2500 patients, of whom 1000 received SAVR between 1987 and 2007. Of these early group, 186 (18.6%) were octogenarians [4]. The remaining 495 octogenarians were a part of a later group 1500 patients (33.0%) who received SAVR between 2008 and 2017 [52]. The advice for reimbursement policy might be held responsible for this effect on age. Age is also an important component in the calculation of risk scores. A mix of preoperative risk scores was used in the included series, which allows comparison of temporal trends only within series but not between series. In particular, the older scores such as the logistic Euroscore tend to overestimate the risk of SAVR [3,16]. There is no uniformity in the observed trends. An increase in risk score of patients referred for SAVR after the introduction of TAVI indicates that this procedure does not “absorb” all high-risk patients.

Only 12 authors analyzed the temporal pattern for the type of valve prosthesis in patients undergoing SAVR, and 11 of these showed a decrease in the use of mechanical heart valve prosthesis (MHV) over time. However, there was a continuous decline in the use of MHV before the introduction of TAVI [33]. The younger age group was mostly driving this decrease [22,41]. A decrease in age of patients in whom a BHV was used was noted [1,30,40,42]. However, in these patients, a second procedure must be anticipated [15] because of the risk for structural valve degeneration [13]. Anti-calcification treatment might improve durability [33], but this is still unproven [18,23,30,42]. A future valve-in-valve TAVI could be considered [18,22,25,41,42,53], but its long-term results are still unknown [54]. Valve size matters in these cases: valve-in-valve TAVI using small prosthesis size resulted in a higher 1-year mortality, compared to larger sizes. Moreover, surgeons tended to implant more larger-size valves in high-volume centers [22,42]. This is important since severe patient–prosthesis mismatch is a major determinant of structural valve degeneration. Valve-in-valve procedures have specific problems such as residual high transvalvular gradients and complexity of the procedure. The “landing zone” is easily identifiable in some types of prostheses, and the need for pacemaker implants, or paravalvular leaks, are less frequent [42]. Re-operative SAVR has not reduced with the introduction of the valve-in-valve procedure. It is an accepted option with a good outcome [23] in patients having an unsuitable anatomy for valve-in-valve procedures [42].

Mortality after SAVR decreased after the introduction of TAVI in most series. This can be expected if TAVI “absorbs” the high-risk patients. There are three observations concerning mortality after SAVR: (a) the outcome was better after the use of BHV compared to MHV [26,33]; (b) there was a gender effect: female patients fared worse after SAVR but benefited from TAVI [3]; and (c) patients with the highest risk score showed the greatest absolute reduction in mortality over time [37]. With adjustments for risk, this improvement in outcome is due to better surgical performance [23] and advances in techniques [25]. However, not all risk factors can be included in classic risk score systems. These factors include calcified aortic annulus and porcelain aorta, and a patent LIMA adhering to the sternum. Recalibrating these scores might be necessary [16,20,37,38].

There are some important aspects that need consideration concerning the current observations. First, the results of surgery improved with diversion of high-risk patients to TAVI, and hence better patient selection and reduced time on the waiting list [20,28,32], but also with continued improvement in operative technique and perioperative care, with lower cross-clamp and bypass times, better management of small aortic annuli, adequate cardioplegic solutions, less blood loss, and a lower need for transfusion. Second, in the first few years after its introduction, TAVI was complementary to SAVR because the procedure was performed mainly in patients in whom the operative risk was deemed too high [21]. This has broadened eligibility for any aortic valve procedure rather than displacing SAVR as an alternative treatment [35]. In later years, TAVI was a substitute for SAVR to some degree, since its use expanded into patients with medium and even low risk [11,17,18,21]. This tendency might be enhanced by increased experience of interventional cardiologists, innovations such as periprocedural imaging, smaller delivery catheters, improved devices, use of sedation instead of narcosis, and consequently a decrease in typical problems related to TAVI, such as vascular access problems, stroke, need for a permanent pacemaker, and paravalvular leaks [14]. SAVR and TAVI could become competing modalities to treat patients with aortic valve stenosis. Third, an opposite trend can also be conceived: the availability of TAVI in a center might bring in patients who would otherwise not see a cardiac surgeon or a heart team for a more comprehensive evaluation of the valve disease. This could especially apply for older patients, deemed inappropriately inoperable, with lower ejection fraction and comorbid conditions. This, in turn, could lead to a reduction in unoperated patients [36]. The increase in referral of older patients for SAVR as a response to the initiation of a TAVI program has been labeled as a “halo effect” [16,24,27,37,53]. Increased awareness of patients for several options to treat the diseased aortic valve can play a role in it [20,32,41,44]. The relaxation of criteria for SAVR by the surgeon [2], or a “cross-over” from the TAVI program to SAVR [15,32], might strengthen this trend. Indeed, some series show a positive impact of TAVI on SAVR volume, because in some cases, patients referred for TAVI finally underwent SAVR [55,56]. Still, patients might not undergo an aortic valve procedure in any form. This can be due to a decision of the patient, a perceived high operative risk, presence of comorbid conditions, advanced age, aortic stenosis not being perceived as serious, denial of the severity of symptoms, symptoms attributed to other conditions, or just “becoming old”. Seeking a second opinion (with consequently loss of follow-up) and insurance problems can add to this problem. It seems that with the introduction of TAVI, these reasons did not alter [36]. However, there is still room for expansion of treating a stenotic aortic valve: 40% to 50% of the patients with severe aortic valve stenosis did not undergo SAVR [46,54], while most of them fulfilled the criteria to undergo surgery. Over half of these patients were not evaluated by a cardiac surgeon. Moreover, a large part of these patients did not receive TAVI [54]. This therapeutic gap illustrates the necessity of a comprehensive approach by a dedicated heart team [51]. Many cardiac trials require involvement of such a team [19]. Adequate triage could increase access to valve procedures and reduce time on waiting lists. It could take into account more complex heart disease (concomitant coronary artery disease and involvement of other valves) and non-elective cases [57].

Meticulous assessment of individual patients by a multidisciplinary heart team ultimately leads to aortic valve implantation in most patients with AS considered as high surgical risk [3,19,34]. This team should operate in high-volume centers [48] and should assess the limiting comorbid non-cardiovascular conditions, physical and mental frailty, possibility for independent living, cardiovascular complexity such as coronary and peripheral artery disease, other valve conditions [47,53,54], pulmonary artery hypertension, prior radiotherapy of the chest, and economic factors [48]. This includes the decision for palliative care if the expected outcome is too poor [47,54]. This approach can improve results by proper patient selection, use of radiologic facilities, hybrid operation rooms, procedure and access selection, improved consistency of treatment, and sharing experiences between operators [19]. Continuous improvements in prosthesis design and selection and implantation technique will increasingly support the role of TAVI [47].

The heart team should consist of interventional cardiologists, imaging experts, experienced surgeons, anesthesiologists, and a clinical coordinator [51,54,58]. Decision making is a shared role [54] and the role of the surgeon must be defined. In this respect, the Centers for Medicare & Medicaid Services [59] made the following statement “Two cardiac surgeons have independently examined the patient face-to-face and evaluated the patient’s suitability for open aortic valve replacement surgery; and both surgeons have documented the rationale for their clinical judgment and the rationale is available to the heart team. The patient preoperatively and postoperatively is under the care of a heart team: a cohesive, multidisciplinary team of medical professionals. The heart team concept embodies collaboration and dedication across medical specialties to offer optimal patient-centered care. The heart team’s interventional cardiologist(s) and cardiac surgeon(s) must jointly participate in the intra-operative technical aspects of TAVR”. This works well [51]. The surgeon should develop as a valve expert with experience in open surgery for complex cases, minimally invasive surgery, and endovascular techniques. This can be done using standardized, formal, and structured postgraduate training with certification, under joint supervision by an interventional cardiologist and cardiac surgeon, and sponsored by scientific societies and industry. This training should include understanding equipment and device functioning, obtaining vascular access and closure, valve crossing, delivery and deployment of the device, cardiac imaging during the procedure, bailout strategies, and cerebral protection [60]. Surgeons may also be helpful as gatekeepers for TAVI, since non-expert caregivers might overestimate the operative risk [20]. Hurdles might be the willingness of the cardiologists to “share the room”, the lack of surgical manpower or funding, and the availability of high-volume centers [60]. Since TAVI started as a procedure having a need for general anesthesia and surgical access for delivery of the device, surgeons already played a role in the procedure, especially for complications that required surgical attendance. Although general anesthesia is less needed and the procedure can be performed percutaneously because of smaller devices, an electronic survey in 2016 revealed that surgeons still play a role: a majority of the surgeons were involved in the pre-, intra-, and post-operative care for patients undergoing TAVI. Over 50% performed technical aspects such as access, alternative access, insertion, pacing, valve crossing, delivery sheath, dilatation, position and deployment of the valve, operating imaging equipment, removal and closure, or open repair [51]. This is much more than a supportive role [53].

## 5. Future Directions and Conclusions

Depending on the expected referrals for SAVR and TAVI, there is a need for training of surgeons and interventional cardiologists and other dedicated personnel for both approaches. Individual risk evaluation and patient selection for either treatment by a heart team consisting of interventional cardiologists and cardiac surgeons should be advocated [3,19,34]. Many cardiac trials require involvement of such a team [19]. Heart teams can improve results by improved patient selection, use of radiologic facilities, hybrid operation rooms, procedure and access selection, improved consistency of treatment, and sharing experiences between operators [19]. Surgeons may also be helpful as gatekeepers for TAVI, since non-expert caregivers might overestimate the operative risk [20]. Valve-in-valve procedures could be further developed.

It can be concluded that the introduction of TAVI has altered the referral of patients for SAVR in an undecided way. The same observation applies for age, risk scores, and outcome, although a trend for lower age, risk score, and short-term mortality can be discerned, especially in hospitals with a large TAVI volume. Confounding and yet unknown factors are the reimbursement policy for TAVI, and the effect of a dedicated heart team on the observed trends.

## 6. Strengths and Limitations

Many included articles are national or regionwide surveys relying on ICD coding, with all potential coding errors and limited data. Some relevant variables remain unmeasured. Participation of hospitals in nationwide surveys can be on a voluntary basis and can be incomplete. These databases are usually restricted to the index hospitalizations. Patients can be lost because of a “second opinion” and subsequent treatment in another hospital or because of migration. Patients’ inclusion might not always be consecutive. Risk scores, if given, are not uniform. Sometimes, surrogates are given, such as the Charlson comorbidity index, or as a listing of separate comorbidities. The older scores tend to overestimate mortality in a significant way. If risk scores are not reported, crude mortality rates are difficult to compare. The early mortality can be shielded by transfer to a nursing home facility. Observational studies are not explanatory; only associations can be detected. Some monocentric series are more detailed but patient series are small and are not necessarily generalizable. Many single-center patient series have also a limited sample size. Some factors, such as improvement in operative techniques or perioperative care for SAVR or increased experience of interventional cardiologists and improved valve designs and delivery systems, have been invoked as possible factors, but are not measured. There are differences across countries concerning healthcare systems in terms of access, cost, and reimbursement for TAVI. These factors can significantly affect the results but are rarely measured. The eras under study sometimes differ. Series with isolated SAVR or combined with CABG are pooled. However, this might cloud the issue; the debate regarding TAVI with PCI and its timing versus SAVR + CABG is still ongoing. Observed time relations are not necessarily causal.

## Figures and Tables

**Table 1:** Authors with publication year, start and end of inclusion, and type of intervention.

Author and Year	Population	Start	TAVI	End	Intervention
Akintoye et al., 2020 [13]	nationwide	2001	2011	2016	SAVR ± CABG
Attias et al., 2015 [14]	mono	2008	2009	2011	iSAVR
Brown et al., 2009 [23]	nationwide	1997	not stated	2006	iSAVR
Brennan et al., 2014 [24]	nationwide	2008	2011	2013	SAVR ± CABG
Chahine et al., 2022 [15]	mono	2011	2012	2020	iSAVR
Cheng et al., 2021 [25]	regionwide	2001	not stated	2018	SAVR ± CABG
Culler et al., 2018 [26]	nationwide	2009	2012	2015	iSAVR
Davies et al., 2016 [27]	mono	2009	2011	2014	iSAVR
Debacker et al., 2016 [1]	regionwide	2005	2008	2015	SAVR ± CABG
Dimagli et al., 2020 [28]	mono	2000	2009	2017	SAVR ± CABG
D’Onofrio et al., 2013 [29]	3 centers	2005	2007	2011	SAVR + TAVI
Dunning et al., 2011 [30]	nationwide	2004	not stated	2009	SAVR ± CABG
Englum et al., 2016 [16]	mono	2006	2011	2013	SAVR ± CABG
Gaede et al., 2017 [31]	nationwide	2012	2008	2015	iSAVR
Gandjian et al., 2022 [17]	mono	2012	2015	2018	iSAVR
Grant et al., 2010 [32]	mono	2006	2008	2009	SAVR ± CABG
Guimaron et al., 2021 [18]	nationwide	2007	2012	2018	iSAVR
Heinze et al., 2015 [3]	mono	2007	2009	2011	iSAVR in female
IBID	IBID	IBID	IBID	IBID	iSAVR in male
Jimenez et al., 2019 [33]	nationwide	2001	2008	2015	SAVR ± CABG
Jones et al., 2019 [19]	regionwide	2006	2008	2016	iSAVR
Khounlaboud et al., 2015 [34]	mono	2006	2008	2014	iSAVR
Kundi et al., 2018 [35]	nationwide	2011	>1 year	2014	iSAVR (no-TAVI hosp)
IBID	IBID	IBID	no TAVI	IBID	iSAVR (TAVI hosp)
Malaisrie et al., 2011 [36]	mono	2006	2008	2009	SAVR ± CABG
Martin et al., 2015 [20]	mono	2003	2007	2013	iSAVR
Maximus et al., 2018 [44]	regional	2009	2011–2014	2014	iSAVR
IBID	IBID	IBID	IBID	IBID	SAVR + CABG
Mori et al., 2019 [37]	mono	2011	2011	2016	iSAVR
Mullan et al., 2020 [38]	nationwide	2005	not stated	2014	iSAVR
IBID	IBID	IBID	IBID	IBID	SAVR + CABG
Nguyen et al., 2017 [39]	3 centers	2011	2011	2014	iSAVR
IBID	IBID	IBID	2011	IBID	Mi-SAVR
Nguyen et al., 2022 [21]	nationwide	2007	2009	2019	iSAVR
IBID	IBID	IBID	IBID	IBID	SAVR + CABG
Reinöhl et al., 2015 [40]	nationwide	2007	2007	2013	iSAVR
Si et al., 2019 [41]	nationwide	2002	2008	2015	iSAVR
IBID	IBID	IBID	IBID	IBID	SAVR + CABG
Silashi et al., 2016 [42]	mono	2002	2007	2012	SAVR ± CABG
Siregar et al., 2014 [43]	nationwide	1995	not stated	2010	SAVR ± CABG
Tam et al., 2020 [22]	nationwide	2004	2008	2016	iSAVR
Wang et al., 2014 [2]	mono	2008	2011	2012	iSAVR

CABG: coronary artery bypass graft, iSAVR: isolated surgical aortic valve replacement; SAVR ± CABG: SAVR with or without CABG. IBID: refers to the same series as above.

**Table 2:** The volumes, patient age, and mortality at the start and the end of the inclusion.

Author Year	Volume 1	Volume 2	Age 1	Age 2	Mort 1	Mort 2
Akintoye et al., 2020 [13]	49,357	10,050	**67.6 ± 13.5**	**65.5 ± 12.8**	**5.4%**	**2.7%**
Attias et al., 2015 [14]	18	65	**78.5 ± 6.5**	**85.3 ± 5.9**	22.0%	13.8%
Brown et al., 2009 [23]	9407/y	15,397/y	**65.9**	**67.9**	**3.4%**	**2.6%**
Brennan et al., 2014 [24]	36,141	46,239	72 (64–79)	73 (64–80)	**3.4%**	**2.5%**
Chahine et al., 2022 [15]	130	134	**74.0**	**67.5**	1.5%	2.2%
Cheng et al., 2021 [25]	768/y	1048/y			**4.39%**	**1.89%**
Culler et al., 2018 [26]	22,076	49,362	grouped	grouped	**5.16%**	**3.67%**
Davies et al., 2016 [27]	215	163				
Debacker et al., 2016 [1]	319	343	74	73		
Dimagli et al., 2020 [28]	615	1292	**grouped**	**grouped**	**2.9%**	**0.7%**
D’Onofrio et al., 2013 [29]	395	1000	**76.8 ± 6.7**	**78.2 ± 7.8**	2.8%	3.4%
Dunning et al., 2011 [30]	7396	9333	**68.9**	**69.5**	**4.4%**	**3.9%**
Englum et al., 2016 [16]	505	545	**69**	**74**	2.8%	1.5%
Gaede et al., 2017 [31]	10,100	9502			2.9%	2.9%
Gandjian et al., 2022 [17]	28,000	27,000	81	80	**2.9%**	**1.0%**
Grant et al., 2010 [32]	174	253	71 (62–77)	72 (63–78)	2.9%	2.1%
Guimaron et al., 2021 [18]	8819	18,579	**75**	**74**	**2.34%**	**1.22%**
Heinze et al., 2015 [3]	90	67	**75.6 ± 8.5**	**71.6 ± 9.0**	7.8%	3%
IBID	101	99	68.6 ± 11.2	68.1 ± 9.0	1%	0%
Jimenez et al., 2019 [33]	7384	19,649	**73.2**	**75.1**	**7.45%**	**5.01%**
Jones et al., 2019 [19]	191	514	**74 (66–80)**	**77 (69–83)**		
Khounlaboud et al., 2015 [34]	229	288	83.2 ± 2.0	83.5 ± 2.1	5.2%	4.3%
Kundi et al., 2018 [35]	3708	4286	**78.1 ± 7.4**	**75.6 ± 6.4**	**4.1%**	**3.4%**
IBID	5762	5001	**IBID**	**IBID**	**4.0%**	**2.4%**
Malaisrie et al., 2011 [36]	362	497	78 (67–83)	78 (68–85)	2.8%	3.2%
Martin et al., 2015 [20]	529	1064	68.3 ± 10.8	69.1 ± 10.2	**3.6%**	**1.8%**
Maximus et al., 2018 [44]	3111	3430			3.4%	2.4%
IBID	1929	1755			5.1%	6.0%
Mori et al., 2019 [37]	80	80			1.3%	2.1%
Mullan et al., 2020 [38]	8/100,000	12/100,000	65	67	**2.5%**	**2.0%**
IBID	7/100,000	7/100,000	72	72	**5.2%**	**4.8%**
Nguyen et al., 2017 [39]	171	210	as curve	as curve	1.8%	0.9%
IBID	145	227	IBID	IBID	0.6%	0.1%
Nguyen et al., 2022 [21]	7616	6896	**71 ± 1**	**68 ± 9**	**4.3%**	**1.1%**
IBID	3267	3182	**74 ± 8**	**71 ± 8**	**7.2%**	**1.9%**
Reinöhl et al., 2015 [40]	8622	7048	as curve	as curve	**3.8%**	**2.2%**
Si et al., 2019 [41]	1232	1697	70.2 ± 11.4	71.0 ± 11.0	2.8%	2.0%
IBID	1388	1503	74.0 ± 7.8	74.5 ± 8.0	4.5%	3.7%
Silashi et al., 2016 [42]	139/y	322/y	**69.7 ± 12.1**	**70.2 ± 9.4**		
Siregar et al., 2014 [43]	11/100,000	27/100,000	**66.3 ± 11.5**	**70.2 ± 10.8**	3.5%	2.4%
Tam et al., 2020 [22]	41,099	173,291	**67.1**	**68.6**		
Wang et al., 2014 [2]	35/3 y	33/2 y	**82.3**	**84.1**	0%	0%

## Data Availability

Not applicable.
